# *A priori *postulated and real power in cluster randomized trials: mind the gap

**DOI:** 10.1186/1471-2288-5-25

**Published:** 2005-08-18

**Authors:** Lydia Guittet, Bruno Giraudeau, Philippe Ravaud

**Affiliations:** 1 Département d'Epidémiologie, Biostatistique et Recherche Clinique, Groupe Hospitalier Bichat-Claude Bernard (AP-HP) – Université Paris 7, Paris, France; 2 INSERM U 738, Université Paris 7, Paris, France; 3 INSERM CIC 202, Faculté de Médecine, Université François Rabelais, Tours, France; 4 INSERM U 717, Université Paris 7, Paris, France

## Abstract

**Background:**

Cluster randomization design is increasingly used for the evaluation of health-care, screening or educational interventions. The intraclass correlation coefficient (ICC) defines the clustering effect and be specified during planning. The aim of this work is to study the influence of the ICC on power in cluster randomized trials.

**Methods:**

Power contour graphs were drawn to illustrate the loss in power induced by an underestimation of the ICC when planning trials. We also derived the maximum achievable power given a specified ICC.

**Results:**

The magnitude of the ICC can have a major impact on power, and with low numbers of clusters, 80% power may not be achievable.

**Conclusion:**

Underestimating the ICC during planning cluster randomized trials can lead to a seriously underpowered trial. Publication of *a priori *postulated and *a posteriori *estimated ICCs is necessary for a more objective reading: negative trial results may be the consequence of a loss of power due to a mis-specification of the ICC.

## Background

A cluster randomized trial involves randomizing social units or clusters of individuals, rather than the individuals themselves. This design, which is increasingly used for evaluating health-care, screening and educational interventions [1-3], presents specific constraints that must be considered during planning and analysis [4,5].

The responses of individuals within a cluster tend to be more similar than those of individuals of different clusters. This correlation leads to an increased required sample size in randomized trials of clusters compared with that of individuals, although this clustering effect is rarely taken into account. Thus, in a recent review of cluster randomized trials in primary care, Eldridge *et al *[6] reported that only 20% of studies accounted for clustering in the sample size calculation. Similar results were found in other reviews, as listed by Bland [7]. The increase in sample size is measured through an inflation factor, which is a function of both the cluster size and the intraclass correlation coefficient (ICC), which appraises the correlation between individuals within the same cluster [1-3,8]. Therefore an *a priori *value for this correlation must be postulated during planning. However, estimates of this correlation are rarely available, and, if available, are often uncertain. Indeed the correlation would differ according to outcome, setting, intervention, covariate adjustment and also sampling [5,9,10]. Therefore, a discrepancy between *a priori *postulated and *a posteriori *estimated ICCs may occur.

The discrepancy between *a priori *postulated and *a posteriori *estimated ICCs may be reduced by intermediate estimation of the ICC, thus allowing a re-estimation of the required sample size [11]. However, the room to manœuvre to increase the sample size may be restricted. Indeed, including new clusters may be difficult, either because the number of clusters is limited [10,12-15] (which may occur when the randomization unit is defined by a geographic area or hospital, for example) or because clusters are frequently randomized all at once and not one at a time. The cluster size itself may also be limited (e.g., by the size of a family or because the number of patients followed up in a clinical practice cannot be increased [16]), which then disallows the increase in sample size by increasing cluster size.

The purpose of our study was to assess the consequence on power of the ICC and to what extent the discrepancy between *a priori *postulated and *a posteriori *estimated ICCs may induce a loss in power in cluster randomized trials.

## Methods

We considered a completely cluster randomized design with a continuous outcome (normally distributed) measured at a single time point. We assumed an equal number of clusters randomized to each arm and a fixed common cluster size. The sample size is calculated as follows [1]:



where *m *is the cluster size, *g *is the number of clusters per arm, *ρ *is the ICC, *ES *is the effect size (defined as ratio between the absolute difference between the two intervention-specific means (|Δ|) and the standard deviation (*σ*)) and *z*_1-*α*/2 _and *z*_1-*β *_are the critical values of the standard normal distribution corresponding to error rates *α *(two-sided) and *β*, respectively. One recognizes the sample size calculation for an individually randomized trial inflated by a factor equal to [1 + (*m *- 1)*ρ*] defined as the variance inflation factor. When the cluster size varies, *m *refers to the average cluster size.

### Power contour graphs

To quantify the influence of the ICC on the power, we drew two kinds of power contour graphs. First, considering an effect size and an a *priori *postulated ICC, we considered several combinations of numbers of clusters and cluster sizes that allow for achieving 80% power. Then considering these combinations, we plotted the real power as a function of the ICC, which may differ from the *a priori *postulated value. Two values of *a priori *postulated ICC (0.005, 0.02) and five numbers of clusters per intervention arm (3, 5, 10, 20 and 40) were considered for these graphs. The effect size was fixed at 0.25.

We also drew power contour graphs, showing combinations of cluster sizes and number of clusters leading to a pre-specified power, with type I error fixed at 5%. Four power levels were considered (90, 80, 60 and 40%), 3 effect sizes (0.25, 0.50 and 0.75) and 4 levels of *ρ *(0.005, 0.020, 0.050 and 0.100). These ICC values were chosen according to previously published estimates [3,6,12,16-23].

### Maximal theoretical achievable power

We determined the maximal achievable power given a limited number of randomized clusters (i.e., considering an infinite cluster size) or a limited cluster size (i.e., considering an infinite number of clusters). For a limited number of clusters, results were graphically illustrated by considering 5 numbers of clusters per intervention arm (3, 5, 10, 20 and 40) for 2 effect sizes (0.25, 0.5).

## Results

### Influence of the discrepancy between a priori postulated and a posteriori estimated ICCs on power

**Figure 1:**
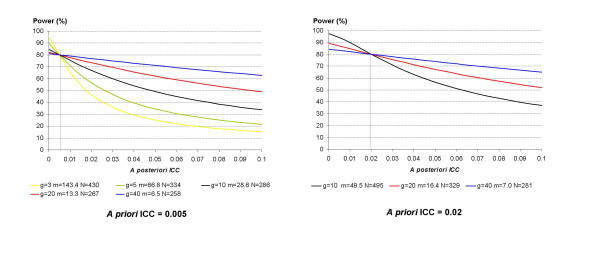
Real power of cluster randomized trials according to the discrepancy between the *a priori *postulated and *a posteriori *estimated intraclass correlation coefficients. The effect size to be detected is fixed at 0.25 and power at 80%. *g *is the number of clusters per arm, *m *is the average cluster size and *N *is the total number per intervention arm considering an *a priori *postulated ICC of 0.005 or 0.02.

Figure 1 displays the real power associated with a study whose *a posteriori *estimated ICC would differ from the *a priori *postulated one. With an *a priori *ICC of 0.02, as few as 5 clusters per intervention arm is not enough to achieve a power of 80% to detect an effect size of 0.25. Power decreases as the ICC increases, and the loss is all the more important when the number of clusters is small. For example, if the *a priori *ICC was fixed at 0.005 and the *a posteriori *ICC is as high as 0.01, the power falls to 70.8% with 5 clusters per intervention arm, instead of the targeted 80% power, whereas the power is almost safeguarded with 20 clusters per intervention arm (real power 77.7%).

**Figure 2:**
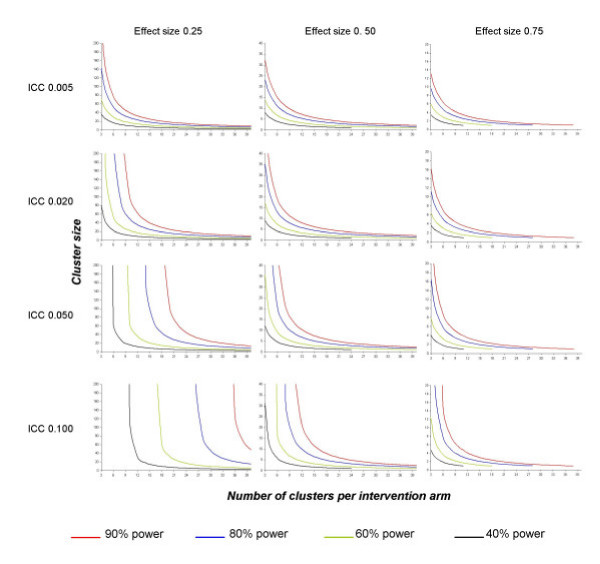
Power contour graphs for several intraclass correlation coefficients (ICCs) and effect sizes*. Effect size is presented in columns and ICC in rows. In situations above or to the right of the red curve, the statistical power is greater than 90%. In situations between the red and blue curves, the statistical power is between 80% and 90%. In situations between the blue and red curves, the statistical power is between 60% and 80%. For vertical curves, increasing the cluster size is pointless. The number of subjects required, assuming individual randomization, is 24 to achieve a power of 40% to detect an effect size of 0.50, and 38, 28, 18 and 11 to achieve powers of 90%, 80%, 60% and 40%, respectively, to detect an effect size of 0.75, thus, the reason why curves are truncated. *Effect size = absolute difference between the two intervention-specific means divided by the S.D. of the response variable.

Figure 2 displays power contour graphs for combinations of numbers of clusters and cluster sizes. First, let us consider the situation of a fixed number of clusters. In many situations, even a slight increase in the ICC has a great influence on power and leads to a major increase in the required cluster size to keep the desired power. In some situations, even reaching the required power may no longer be possible: the power contour curves tend to be infinite. For instance, assuming that 15 clusters are randomized to each arm and we want to detect an effect size of 0.25 with 80% power, we would need an average cluster size of 25 patients with an *a priori *ICC fixed at 0.02. To keep 80% power, the required mean cluster size should be increased to 98 for an ICC of 0.05, which represents 1095 more subjects per arm. If the ICC actually equals 0.10, 80% power is no longer achievable without recruiting additional clusters. The phenomenon is all the more acute when the number of fixed clusters is low.

Second, when the mean cluster size is limited but the number of clusters is not, an increase in the ICC may also be of great consequence. As an example, considering a mean cluster size of 100, we would need to randomize 8 clusters per arm to detect a 0.25 effect size with 80% power when the ICC is fixed at 0.02. This number of clusters is raised to 15 and 28 when the ICC is fixed at 0.05 and 0.10, respectively, or 700 and 2000 additional subjects, respectively, per arm.

### Maximal theoretical power with infinite cluster size

In a cluster randomized trial aimed at detecting an effect size *ES *at a pre-specified *α *level with an *a priori *postulated ICC equal to *ρ*, changing the cluster size *m *and/or the number of clusters *g *per group changes *β *and therefore power. Power is thus related to the *f*(*m*, *g*) = (*z*_1-*α*/2 _+ *z*_1-*β*_)^2 ^function defined as



**Figure 3:**
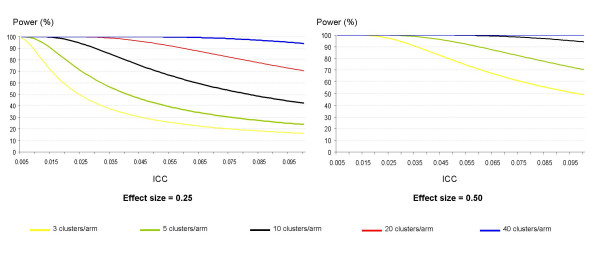
Theoretical maximal power assuming an infinite cluster size for several fixed numbers of clusters according to two different effect sizes *. *Effect size = absolute difference between the two intervention-specific means divided by the S.D. of the response variable.

When *m*, the mean cluster size, tends to be infinite, *f*(*m*, *g*) tends to be an asymptotic value, but there is no limit when *g*, the number of clusters, is infinite:



Therefore, although power is not theoretically limited when the number of clusters can be increased, a maximal reachable power is possible when this number is fixed and only the cluster size can be increased. This maximum theoretical power is defined as:



where Φ^-1^( ) refers to the inverse cumulative function associated with the standard normal distribution. This maximal theoretical power decreases when the ICC increases and/or the number of clusters decreases (Figure 3). In some cases, an 80% or 90% power is not achievable even with a theoretical situation of infinite cluster sizes. Thus, when 5 clusters are randomized to each arm, a power of 80% to detect an effect size of 0.50 cannot be achieved if the ICC is greater than 0.079 (and this limit equals 0.058 when 90% power is considered). For an effect size of 0.25, this upper ICC limit is 0.019 for 80% power and 0.014 for 90% power.

## Discussion

The ICC is a nuisance parameter that has to be *a priori *specified when planning a cluster randomized trial. The magnitude of this coefficient has a major impact on power, particularly with a small number of randomized clusters. Our results were derived considering a continuous outcome, but in their simulation study, Donner and Klar [24] showed that power never differs from more than one percentage point in continuous or binary outcomes. Moreover, we did not take into account any potential variability in cluster size, which is already known to reduce power [25]. When planning cluster randomized trials, variability in cluster size is rarely taken into account, and the cluster size *m *is generally replaced by the mean cluster size. An underestimation of the ICC may therefore be expected to have similar consequences when cluster size is constant. In the end, an underestimation of the ICC during planning could therefore lead to a severely underpowered study and thus questionable results.

In cluster randomized trials, it is known that for a fixed total number of subjects, the higher the number of clusters (and thus the smaller the average cluster size), the higher the power [2,4,5,14,24,26,27]. In the extreme case, in clusters of size one, individuals are randomized, with no loss of power because of correlation between subjects. Moreover, it has also been shown that increasing cluster size improves the power up to a certain threshold, which depends on the value of the ICC [24,27]. Therefore, when planning a cluster randomized trial, the optimal strategy is indeed to randomize a large number of clusters [1,2,12,29]. Such a strategy first allows for decreasing the total sample size for a pre-specified power and second, as our results show, protects against a loss of power induced by an underestimation of the ICC when planning. However, because of logistic constraints, the number of randomized clusters may be limited, and indeed, the review by Eldridge *et al *[6] noted that half of the cluster randomized trials analyzed had fewer than 29 clusters in each arm. Therefore, for most cluster randomized trials, the *a priori *postulated value of the ICC has a great impact on power.

When planning trials, the *a priori *postulated ICC will rarely be very reliable. During the study, an intermediate estimation of the ICC can be assessed, thus allowing a sample size adjustment [11]. But the determination of this intermediate estimation is not without error, as was shown in the study by Moore et al [28], in which the intermediate ICC was 0.012 and the final one 0.031. A sensitivity analysis must therefore be undertaken when planning, to account for uncertainty of the ICC. In the extreme situations, when very few clusters can be randomized, such a sensitivity analysis may illustrate the high risk of performing an underpowered study and thus highlight arguments for not performing the study.

When reporting the study results, investigators should publish both the ICC used during the planning and the *a posteriori *estimated one, as recommended initially by some authors and recently by the extension of the CONSORT statement for cluster randomized trials [27,29-31]. However, such information is rarely available. We studied cluster randomized trials published between January 2003 and December 2004 in the *British Medical Journal*, "which contains more such reports than any other journal" [7], and the published extension of the CONSORT statement [30]). Of 16 published studies, 5 (31.2%) did not report an *a priori *postulated ICC and 2 reported no sample size calculation. Only 5 (31.2%) reports provided *a posteriori *estimated ICCs (without any confidence intervals). Such under-reporting disallows assessing the discrepancy between the *a priori *postulated ICC and the *a posteriori *estimated one. However, reporting both ICCs would help readers "assess the appropriateness of the original sample size calculations as well as the magnitude of the clustering for each outcome" [30] and help investigators design future trials [1,27,31]. It would also help readers understand trial results, particularly negative ones: a study may prove to be negative just by a loss of power induced by an *a priori *underestimation of the ICC. On a formal point, the publication format of the *a posteriori *estimated ICC should follow the recommendation by Campbell et al., who advocate specifying a description of the data set and information on the method used to assess it and the precision of the estimate [32].

In conclusion, our study supports modifications in investigators' practices when planning trials and reporting results, taking into account the uncertainty of the ICC by favoring a high number of clusters and publishing this parameter. For readers, an objective reading of trial results, particularly negative results, requires knowledge of *a priori *and *a posteriori *estimated ICCs.

## Competing interests

The author(s) declare that they have no competing interests.

## Authors' contributions

This study was designed by LG, BG and PhR. LG performed the statistical analysis and drafted the article, which was then revised by BG and PhR.

## Pre-publication history

The pre-publication history for this paper can be accessed here:


